# The HTAi early HTA definition: a welcome milestone, and what must follow

**DOI:** 10.1017/S0266462325100470

**Published:** 2025-08-26

**Authors:** Christian Suharlim

**Affiliations:** Thermo Fisher Scientific, Waltham, MA, USA

The HTAi Working Group did the field a service. Their consensus-based definition of early health technology assessment (HTA) – an HTA conducted to inform decisions about subsequent development, research, or investment by explicitly evaluating the potential value of a conceptual or actual health technology (1) – offers clarity where there was confusion. Equally important was the inclusive process that brought diverse stakeholders to agreement.

The phrase “to inform decisions” signals a deliberate shift towards a broader view of early HTA. Some earlier definitions framed it as a binary “go or no-go” tool (2) – an oversimplification. Early HTA is not about predicting success, but navigating uncertainty. Many technologies, especially in global health, mature into value over time. A diagnostic overlooked in wealthy countries could be vital elsewhere. Early HTA helps chart where value might lie – and under what conditions it can emerge.

The definition also broadens the audience. Early HTA is not just for industry. Donors and philanthropies use it to assess life-saving technologies in low-resource settings. In some LMICs, governments fund homegrown innovations, using early HTA as a tool of industrial policy. Here, value is defined more by public benefit, equity, and long-term impact than short-term return (3). The working group’s framing of value – “varying by perspective, stakeholder, and decision context” – is a necessary expansion.

Concerns about “inaccuracy” surfaced during the consensus process. The Working Group rightly noted that all HTA involves uncertainty – and early HTA should make that explicit. But the discomfort runs deeper: the real concern is getting it wrong – where imperfect forecasts prematurely shelve promising technologies. Applied narrowly, early HTA could exclude innovations with low commercial appeal but high societal value. These may later serve marginalized populations or enable future innovation. Addressing this requires open dialogue about risk, equity, and who bears the cost of early decisions.

To fulfill its promise, early HTA needs a framework for interpretation – especially when results are negative. A technology that underperforms on technical or cost-effectiveness grounds may still deliver value in the right context or with different actors. Developed by the author, the **SCALE** framework considers: **Stakeholder** (are we considering stakeholders beyond traditional payers and recognizing what they value?), **Contextual fit** (can suboptimal tools have an outsized impact in certain settings?), **Actor alignment** (is the right player leading development?), **Longitudinal potential** (could today’s marginal product be tomorrow’s platform?), and **Effectiveness** (does the technology meet key performance and cost-effectiveness thresholds?). A weak signal in one dimension should not eclipse strong ones in others. Early HTA should widen the aperture, not narrow it.

Finally, early HTA must become more transparent. Many assessments remain inaccessible behind corporate firewalls (4). To inform public decisions or donor priorities, ongoing efforts should continue to improve visibility and replicability. Structured reporting and open dialogue would improve transparency and surface insights that siloed analyses miss. This consensus is a milestone. The next step is to operationalize it by building methods, tools, and platforms to make early HTA practical and impactful. Done well, early HTA can guide investment, support innovation, and promote more equitable, efficient, and high-quality health systems.
